# Normal Appearing and Diffusely Abnormal White Matter in Patients with Multiple Sclerosis Assessed with Quantitative MR

**DOI:** 10.1371/journal.pone.0095161

**Published:** 2014-04-18

**Authors:** Janne West, Anne Aalto, Anders Tisell, Olof Dahlqvist Leinhard, Anne-Marie Landtblom, Örjan Smedby, Peter Lundberg

**Affiliations:** 1 Radiation Physics, Department of Medicine and Health, Linköping University, Linköping, Sweden; 2 Center for Medical Image Science and Visualization, Linköping University, Linköping, Sweden; 3 Department of Neurology, Linköping University, and Neurology, UHL and LiM County Council of Östergötland, Linköping, Sweden; 4 Radiological Sciences, Department of Medicine and Health, Linköping University, Department of Radiation Physics, UHL County Council of Östergötland, Linköping, Sweden; 5 Radiology, Department of Medicine and Health, Linköping University, Department of Radiology, UHL County Council of Östergötland, Linköping, Sweden; Institute Biomedical Research August Pi Sunyer (IDIBAPS) - Hospital Clinic of Barcelona, Spain

## Abstract

Introduction: Magnetic Resonance Imaging is a sensitive technique for detecting white matter (WM) MS lesions, but the relation with clinical disability is low. Because of this, changes in both ‘normal appearing white matter’ (NAWM) and ‘diffusely abnormal white matter’ (DAWM) have been of interest in recent years. MR techniques, including quantitative magnetic resonance imaging (qMRI) and quantitative magnetic resonance spectroscopy (qMRS), have been developed in order to detect and quantify such changes. In this study, qMRI and qMRS were used to investigate NAWM and DAWM in typical MS patients and in MS patients with low number of WM lesions. Patient data were compared to ‘normal white matter’ (NWM) in healthy controls. Methods: QMRI and qMRS measurements were performed on a 1.5 T Philips MR-scanner. 35 patients with clinically definite MS and 20 healthy controls were included. Twenty of the patients fulfilled the ‘Barkhof-Tintoré criteria’ for MS, (‘MRIpos’), whereas 15 showed radiologically atypical findings with few WM lesions (‘MRIneg’). QMRI properties were determined in ROIs of NAWM, DAWM and lesions in the MS groups and of NWM in controls. Descriptive statistical analysis and comparisons were performed. Correlations were calculated between qMRI measurements and (1) clinical parameters and (2) WM metabolite concentrations. Regression analyses were performed with brain parenchyma fraction and MSSS. Results: NAWM in the MRI_neg_ group was significantly different from NAWM in the MRI_pos_ group and NWM. In addition, R1 and R2 of NAWM in the MRI_pos_ group correlated negatively with EDSS and MSSS. DAWM was significantly different from NWM, but similar in the MS groups. N-acetyl aspartate correlated negatively with R1 and R2 in MRI_neg_. R2 of DAWM was associated with BPF. Conclusions: Changes in NAWM and DAWM are independent pathological entities in the disease. The correlation between qMRI and clinical status may shed new light on the clinicoradiological paradox.

## Introduction

Multiple Sclerosis (MS) is often described as a chronic, inflammatory, demyelinating disease of the central nervous system. Magnetic Resonance Imaging (MRI) is a sensitive imaging technique for detecting MS lesions in vivo, and conventional T2-weighted imaging is widely used to monitor and diagnose MS [1]. However, the correlation between focal white matter (WM) lesions and clinical disability is only modest, a phenomenon which has persisted for many years and is referred to as the *clinicoradiological paradox* [2]. One of the factors thought to explain this discrepancy is individual variations in brain plasticity and cortical reorganization, which may limit the clinical disability caused by focal lesions [3], [4], [5]. Because of this, interest in other pathological tissue changes, beside WM lesions, has been of interest in recent years. These include changes in cortical grey matter [6], [7], deep grey matter structures [8], and changes in normal appearing white matter (NAWM) [9], [10], [11], [12]. However, mechanisms leading to persistent disability in MS remain unclear.

In addition to WM lesions and NAWM, diffuse abnormal signal intensity changes are often seen on conventional T2-weighted images, but these are mostly unaccounted for in the radiological criteria for MS [13]. These regions, which have fuzzy borders and signal intensity that is slightly higher than NAWM, but lower than WM lesions, have been referred to as ‘diffusely abnormal white matter’ or alternatively ‘dirty-appearing white matter’ (DAWM) [14], [15], [16], [17], [18]. Several studies have suggested that DAWM may be a separate pathological entity from NAWM and focal WM lesions [15], [17], [18]. DAWM has been suggested to be a chronic process due to Wallerian degeneration, secondary to focal WM lesions [16], and this is consistent with histological findings [19], [20].

Regions of DAWM are difficult to characterize using standard T2-weighted imaging because of their diffuse nature, and NAWM in MS patients is identical to the normal white matter (NWM) in healthy subjects. Recent studies, however, emphasize the importance of these diffuse changes as they may be important markers of disease progression [17], [18]. Technological developments and advanced MR techniques have therefore been suggested for characterizing and quantifying DAWM, as well as for detecting changes in NAWM. These approaches include ‘diffusion tensor imaging’ (DTI) [17], [18], [21], ‘quantitative magnetization transfer imaging’ (qMTI) [15], [18], ‘quantitative magnetic resonance imaging’ (qMRI) [17], [18], [22], and ‘quantitative magnetic resonance spectroscopy’ (qMRS) [23], [24].

QMRI are techniques to obtain the absolute magnetic properties of tissue water, such as the effective longitudinal (T1) and transversal (T2) relaxation times, or their inverses R1 and R2, and the proton density (PD). The determination of these properties using qMRI is in principle insensitive to both MR-scanner hardware and MRI acquisition protocol.

In MS pathology it has been shown that increased water T1 and T2 times (decreased R1 and R2) are linked to increased water contents, caused by oedema, as well as increased extracellular spaces, caused by axonal loss and demyelination [16], [17], [25], [26]. However, in MS lesions a wide range of T1 and T2 values have been reported, probably a consequence of large individual variability in lesion pathology [27], [28]. Several studies on MS also reported elevated T1 values within NAWM (a global shift affecting all WM) [29], [30], [31]. In addition, Seewann *et al.* showed that both T1 and T2 times are also prolonged in DAWM, an observation that is consistent with axonal loss, decreased myelin density and gliosis [17]. In particular in their excellent histopathological post-mortem study they found that DAWM appears to be a chronic process involving the development of an axonal pathology that is different from both NAWM and focal WM lesions, with more pronounced microglial activation than NAWM and absence of acute axonal pathology as is found in lesions. Moreover, Vrenken *et al.* showed that T1 times in DAWM differed between primary-progressive (PP) and secondary-progressive (SP) MS patients [18] and that qMRI characteristics of NAWM changed with distance to focal WM lesions [32]. In combination, these findings suggest that the sensitivity of qMRI may be higher than conventional imaging, and thus of value for describing diffuse pathology. Moreover, qMRI may also be used for calculating ‘brain parenchyma fraction’ (BPF) [33] which has previously been used as a reliable assessment of brain atrophy in MS [34], [35].

In a minority of MS patients, no focal WM lesions at all, or very few lesions, are detected using conventional MRI, also after a long time of disease. These are atypical MS patients that constitute an interesting model for investigating MS pathology in NAWM and DAWM. Our research has taken a particular interest in subgrouping MS patients with atypical features, for example this group of MRI negative MS patients [23], [36]. Results have shown increased concentrations of glutamate and glutamine (tGlx) in NAWM of both patients with no radiologically visible lesions (‘MRI_neg_’) and the typical MS patients (‘MRI_pos_’) compared to healthy controls. Moreover, the MRI_pos_ group showed increased myo-Inositol (mIns) concentrations and decreased total N-acetyl aspartate (tNA) concentration in NAWM compared to both the MRI_neg_ and healthy controls. In contrast, the MRI_neg_ group did not show any significant difference in mIns and tNA concentrations compared to the healthy controls.

In the present prospective study, four research questions were addressed: (**1**) Are there any differences in NAWM and DAWM between MS patients with atypical low-lesion MRI examinations and MS patients with typical WM lesions, and are there differences between any of these two groups and healthy controls? (**2**) Are NAWM metabolite concentrations correlated with qMRI measurements of NAWM? (**3**) Is whole brain atrophy related to the qMRI properties of NAWM and DAWM? (**4**) Is the severity of the MS disease, assessed by ‘Multiple Sclerosis Severity Score’ (MSSS) [37], associated with qMRI measurements of NAWM and DAWM?

## Materials and Methods

### Subjects

A total of 35 clinically definite (CDMS) patients and 20 healthy subjects were included in the investigations. (**i**) Fifteen MS patients with two or fewer T2-hyperintense WM lesions, on a previous clinical examination, were prospectively included in the low-lesion MS group (‘MRI_neg_’), (**ii**) 20 MS patients fulfilling the ‘Barkhof-Tintoré criteria’ as defined in [13] were included in the MS group with typical WM lesions (‘MRI_pos_’), and (**iii**) 20 healthy control subjects were included in the control group (see Table 1). All patients, in both MS groups, fulfilled the Poser criteria: with at least two relapses, separated in space and time [38]. All patients had been confirmed positive for oligoclonal bands in the CSF. The study was approved by The Regional Ethics Committee in Linköping (Dnr: M88-07 T93-08), and written informed consent was obtained from all subjects before study entry.

**Table 1:** Subjects.

	Controls	MRI_pos_	MRI_neg_
Number of subjects	20	20	15
Age, [median (min-max)]	48 (27–72)	46 (20–66)	57 (32–69)
Sex [M/F]	5/15	6/14	1/14
MS type [RR/SP/PP]	N/A	12/7/1	10/3/2
EDSS [Median (min–max)]	N/A	3.25 (1.0–8.5)	2.50 (0.0–6.5)
MSSS [Median (min–max)]	N/A	3.74 (0.45–9.57)	3.65 (0.05–9.38)
Disease duration year [Median (min–max)]	N/A	13 (2–35)	16 (2–44)
Number of MS lesions [Median (min–max)]	N/A	16 (3–30)	**1 (0–20)^†††^**
Brain Parenchyma Fraction (BPF) [Mean±SD]	0.881±0.035	**0.806±0.046** ***	0.868±0.024

***p<0.001 compared to control group (2-sample t-test), ^†††^p<0.001 compared to MRI_pos_ (Mann-Whitney U test). The MS patients were divided in two groups; the MRI_neg_ showed two or fewer T2-hyper-intense WM lesions, on a previous clinical MR examination, whereas the MRI_pos_ fulfilled the ‘Barkhof-Tintoré criteria’ as defined in [13].

### Magnetic Resonance Measurements

Measurements were performed using a 1.5 T Philips Achieva MR-scanner (Philips Healthcare, Best, The Netherlands) using an eight-channel phase array head coil. The qMRI protocol was part of a more extensive investigation including clinical imaging sequences, as well as qMRS measurements.

**Figure 1:**
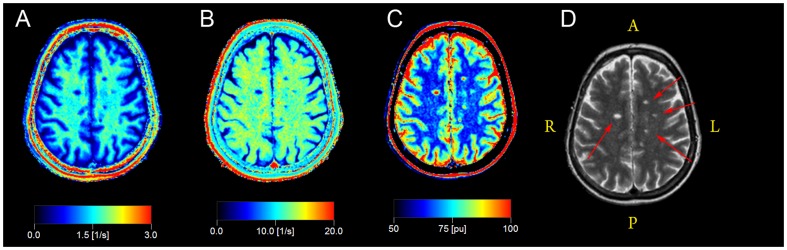
Sample qMRI data maps from a 45-years-old female MRI_pos_ MS patient. a) R1 map, b) R2 map, c) PD map, d) synthetic T2-weighted image is shown for reference. Several partially liquefied lesions are visible (red arrows in d).

QMRI: The multi-slice, multi-echo and multi-saturation delay qMRI method QMAP (also known as QRAPMASTER) was used [22]. For each examination 30 slices were acquired with the qMRI sequence (4.0×0.8×0.8 mm^3^). The QMAP sequence allowed simultaneous quantification of R1 and R2, PD and the local B1 field in a clinically acceptable scanning time. QMAP consists of an interleaved saturation pulse and a Carr-Purcell-Meiboom-Gill sequence (CPMG) acquisition. The saturation, with flip angle θ = 120°, acts on slice *n*, whereas the subsequent acquisition acts on a different slice, *m*. By introducing a shift between slice *n* and slice *m*, a delay was created between the saturation and the acquisition of each particular slice. The sequence was repeated four times, with the shift between *n* and *m* set to 1, 4, 14 and 29. Using 30 slices and TR of 3000 ms, these shifts resulted in saturation delay times (TD) of 100, 400, 1400 and 2900 ms. Each acquisition contained five echoes at TE of 14 ms multiples. The excitation pulse, α, had a flip angle of 90° (X) followed by 180° (Y) refocusing pulses. The refocusing pulses were followed by spoiler gradients. The qMRI sequence resulted in 20 images for each slice; with five echoes for each of the four saturation delays. To accelerate the acquisition, Echo Planar Imaging (EPI) was applied for each echo, using an EPI factor of 3 (gradient spin echo acquisition, GraSE). The R1, R2 and PD maps were retrieved from the raw data using the SyMRI Diagnostics (0.9.3) software (SyntheticMR AB, Sweden, 2011). Sample qMRI maps *from a 45-years-old female MRI_pos_ MS patient* are provided in Fig. 1.

Furthermore, the BPF of each subject was calculated from the qMRI maps, using the approach described in Ref. [33]. This method was based on a partial volume model where brain tissue fractions were estimated from the R1, R2 and PD values. This calculation was also carried out in the SyMRI software.

**Figure 2:**
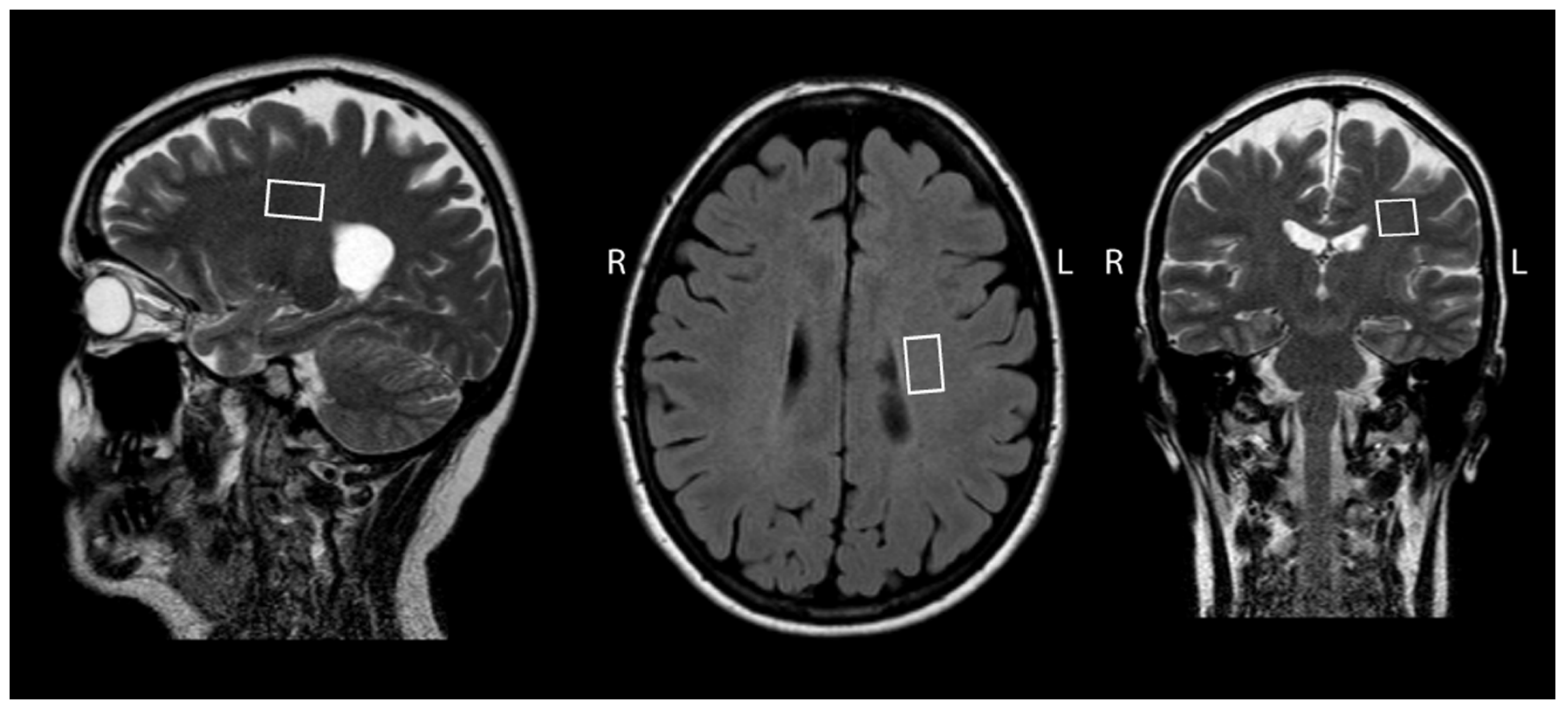
Typical VOI placement in MS patients.

QMRS: Two MRS VOIs were placed bi-laterally in NAWM. The MRS signal was measured using the point-resolved spectroscopy sequence (PRESS), TE = 30 ms, TR = 3 s and 128 transients were averaged. Absolute aqueous fraction concentrations of creatine (tCr-Aq), myo-Inositol (mIns-Aq), glutamate and glutamine (tGlx-Aq) and N-acetyl aspartate (tNA-Aq) were calculated using the procedure described in [24]. Typical MRS VOI placements are shown in Fig. 2. The results of the qMRS measurements was reported in [23] and in this work only correlations to qMRI were considered.

### ROI for Quantitative MR Imaging Analysis

In order to measure brain qMRI values for the different tissue types, standardised ‘Regions of Interest’ (ROIs) were positioned manually in the MS patients and the healthy control group by a radiologist (A.A.). The size of the ROIs was 3×3 voxels (corresponding to 2.4×2.4 mm^2^) and they were placed on synthetic T2-weighted images (TE = 100 ms, TR = 4.5 s), with conventional T2-weighted images used for reference. Synthetic T2-weighted images were generated from the quantitative data using SyMRI Diagnostics (0.9.3), as described elsewhere [22]. Synthetic T2-weighted images were generated from the same data as the qMRI parameter maps and therefore perfectly registered, removing the need for additional data registration.

**Figure 3:**
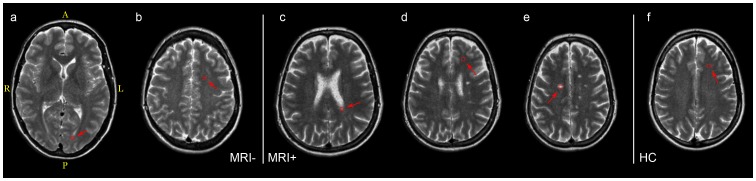
Typical ROI placement in MS patients and healthy controls. a) DAWM in MRI_neg_, b) NAWM in MRI_neg_, c) DAWM in MRI_pos_, d) NAWM in MRI_pos_, e) WM lesion in MRI_pos_, f) NWM in a healthy control. MRI_neg_ subject was a 43 years old female, MRI_pos_ subject was a 66 years old female and healthy subject was a 42 years old female.

In healthy controls, two ROIs were placed in frontal and in parietal NWM in the centrum semiovale (both left and right). Similarly, in MS patients two ROIs were placed in NAWM (left and right), frontally and parietally in the centrum semiovale, making sure that no DAWM or WM lesions were included. Two ROIs were also placed in areas of DAWM, and an additional two ROIs were placed in focal WM lesions, whenever present. The radiological DAWM definition was adapted from [17] and it was defined as a diffuse uniform, non-focal area in the white matter, preferentially periventricular and of increased signal intensity in the T2-weighted image. Compared with the signal intensity of WM lesions, DAWM was observed as subtly, but distinctly increased signal intensity. The DAWM signal intensity decreased towards the border to NAWM, leading to a relatively poorly defined border of DAWM areas, compared to focal WM lesions. Typical ROI placements are shown in Fig. 3. For each of these ROIs, the mean R1, R2 and PD quantitative measurements were obtained.

In addition, the mean qMRI R1, R2 and PD values were also obtained from the MRS VOIs.

### Statistical Analysis

Three different sets of statistical tests were performed using SPSS 21 (SPSS Inc., Chicago, USA). First; descriptive statistics were calculated for qMRI measurements of all tissue types, in all subject groups, and comparisons were also performed using two separate general linear models (GLM1 and GLM2), with ‘subject’ and ‘ROI’ treated as a random effects. In GLM1, the tissue types NAWM, DAWM and WM lesions were compared to NWM in healthy subjects. This was done for each of the two MS groups separately. In GLM2, comparisons were performed between the same tissue types in the two different MS groups (e.g. NAWM in MRI_neg_ compared to NAWM in MRI_pos_).

Second, correlations were calculated between qMRI measurements in NAWM and DAWM, and the clinical parameters (number of lesions, EDSS, MSSS, disease duration, age and BPF). Correlations between qMRI measurements in NAWM and previously reported WM metabolite concentrations in the same subjects (previously reported in [23]) were calculated. In these subjects the mean qMRI values measured inside the MRS VOIs were also correlated with the qMRS measurements of the same VOIs. Pearson correlations were initially used for all variables and Spearman correlations were subsequently used for EDSS and MSSS scores, if significant, to verify findings.

Finally, regression analyses were performed with both MS groups pooled to assess the association between qMRI measurements and BPF, as well as qMRI measurements and MSSS.

The prospective inclusion criteria for the MRI_neg_ group were based on a previous clinical MRI examinations, thus the MRI_neg_ patients could have developed lesions between their previous and the present MRI examination. Therefore, as a control experiment, all tests were subsequently re-evaluated, excluding patients that had developed more than two lesions.

## Results

NAWM, DAWM and WM lesions were present in all MRI_pos_ patients, and NAWM was present in all MRI_neg_ patients. In addition, DAWM was present in twelve of the MRI_neg_ patients, and WM lesions were found in nine of them. No WM lesions at all were observed in six of the MRI_neg_ patients, and DAWM was not observed in three of the MRI_neg_ patients.

**Figure 4:**
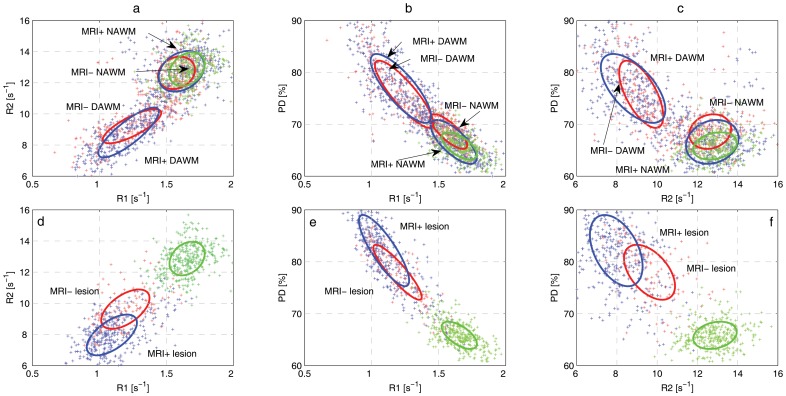
Tissue clusters and in vivo data, in the 3D feature space formed by R1-R2-PD, projected on a,d) R1-R2, b,e) R1-PD and c,f) R2-PD planes. Figures a-c shows NAWM, DAWM and NAWM, figures d-f shows NWM and WM lesions. Cluster ellipses indicates the calculated covariance's of each tissue type separately, red ellipses correspond to the MRI_neg_ group, blue ellipses correspond to the MRI_pos_ group, and the dashed black ellipses correspond to the NWM tissue cluster in healthy controls. The indicated ellipses enclose 95% of the in vivo data.

Descriptive statistics and results of the group comparisons, both comparing MS patients to healthy controls and also comparing MRI_neg_ to MRI_pos_, are presented in Table 2. BPF was significantly lower in the MRI_pos_ group than in the healthy controls, but unaffected in the MRI_neg_ group, see Table 1. In Fig. 4, tissue clusters are displayed in the R1-R2-PD space, showing tissue cluster separation and covariances on top of *in vivo* data. The indicated ellipses enclose 95% of the voxels from each tissue type. The results of correlation analyses between qMRI measurements of all tissue types, in all groups, and clinical parameters are listed in Table 3. Correlations of qMRI and qMRS measurements are listed in Table 4 (qMRS measurements were reported separately in [23] and are not repeated in this paper). Correlations between mean qMRI values measured inside the MRS VOIs and qMRS measurements are reported in Table 5. Finally, the associations between qMRI tissue properties and BPF as well as MSSS are presented in Table 6 and Table 7.

**Table 2:** Descriptive statistics (mean ± SD) and group comparisons for tissue types, in all groups.

	n	R1 [s^−1^]	R2 [s^−1^]	PD [%]
**HC**				
NWM	20	1.65±0.10	12.87±0.66	65.83±1.66
**MRI_pos_**				
NAWM	20	1.61±0.13	12.74±1.01	66.60±3.04
DAWM	20	1.22±0.19***	8.80±1.30***	76.91±5.08***
Lesion	20	1.09±0.11***	7.97±0.91***	82.16±3.78***
**MRI_neg_**				
NAWM	14	1.57±0.10*	12.64±0.66	68.59±2.23***^†^
DAWM	12	1.25±0.15***	9.24±0.70***	75.63±4.21***
Lesion	9	1.18±0.11***	9.46±0.82***^†††^	78.69±3.36***^†^

Stars indicate p-levels of comparisons to NWM in healthy controls and crosses indicate p-levels of comparisons of the same tissue type in MR_pos_- and MRI_neg_ groups.*p<0.05 compared to NWM, **p<0.01 compared to NWM, ***p<0.001 compared to NWM. ^†^p<0.05 compared to same tissue type in MRI_pos_, ^††^p<0.01 compared to same tissue type in MRI_pos_, ^†††^p<0.001 compared to same tissue type in MRI_pos_.

**Table 3:** Pearson correlations between qMRI properties and clinical parameters.

		# Lesions	EDSS	MSSS	DD	Age	BPF
**HC**							
NWM	R1	-	-	-	-	0.023	0.150
	R2	-	-	-	-	0.392	−0.331
	PD	-	-	-	-	0.139	−0.316
**MRI_pos_**							
NAWM	R1	0.108	**−0.483** *	**−0.508** *	−0.135	−0.140	0.399
	R2	−0.273	**−0.550** *	**−0.550** *	−0.128	0.126	0.187
	PD	−0.313	0.191	0.234	−0.028	0.118	−0.357
DAWM	R1	−0.049	0.025	0.222	−0.190	−0.233	0.166
	R2	−0.192	0.005	0.238	−0.253	−0.400	0.292
	PD	−0.003	−0.134	−0.281	0.132	0.121	−0.145
**MRI_neg_**							
NAWM	R1	−0.286	0.285	0.258	0.025	−0.279	−0.074
	R2	0.092	0.352	0.103	0.325	0.375	−0.504
	PD	0.179	−0.161	−0.097	−0.034	0.307	−0.107
DAWM	R1	0.231	0.372	0.409	−0.303	−0.554	0.213
	R2	0.121	0.334	0.446	−0.471	**−0.656** *	0.326
	PD	−0.075	−0.291	−0.276	0.121	0.429	-0.115

*p<0.05, **p<0.01, ***p<0.001.

**Table 4:** Correlations between qMRI properties of NAWM and NWM, in all groups, and qMRS metabolite concentrations.

		tGlx-Aq	tNA-Aq	tCr-Aq	mIns-Aq	tCho-Aq
**HC**						
NWM	R1	−0.161	−0.346	−0.018	0.039	0.195
	R2	−0.301	−0.280	−0.106	0.042	0.254
	PD	0.032	0.205	−0.061	0.112	−0.219
**MRI_pos_**						
NAWM	R1	−0.220	−0.002	−0.197	0.231	**0.446** *
	R2	−0.370	−0.049	−0.229	0.433	0.358
	PD	0.019	−0.028	0.013	−0.059	−0.219
**MRI_neg_**						
NAWM	R1	0.113	−0.334	−0.234	0.069	−0.481
	R2	0.257	−0.279	0.354	0.428	−0.042
	PD	−0.241	0.362	0.454	0.133	0.469

*p<0.05, **p<0.01, ***p<0.001.

**Table 5:** Correlations between mean qMRI parameter values within the larger qMRS VOIs of NAWM and NWM and qMRS metabolite concentrations measured in the same VOIs.

		tGlx-Aq	tNA-Aq	tCr-Aq	mIns-Aq	tCho-Aq
**HC**						
NWM	R1	0.259	0.312	−0.237	−0.444	0.353
	R2	0.150	−0.186	−0.224	**−0.474** *	0.244
	PD	−0.291	0.344	0.320	**0.446** *	−0.354
**MRI_pos_**						
NAWM	R1	−0.351	−0.281	−0.208	0.136	**0.453** *
	R2	−0.441	−0.232	−0.245	0.280	0.260
	PD	0.427	0.107	0.253	−0.078	−0.327
**MRI_neg_**						
NAWM	R1	0.203	**−0.520** *	−0.286	−0.019	0.007
	R2	0.008	**−0.540** *	−0.261	−0.166	0.220
	PD	−0.172	0.442	0.249	0.077	−0.014

*p<0.05, **p<0.01, ***p<0.001.

**Table 6:** Association between qMRI measurements and brain parenchyma fraction (BPF).

		β	SE		p
NAWM	R1	0.378	(0.200)		0.068
	R2	−0.023	(0.016)		0.148
	PD	0.012	(0.007)		0.102
DAWM	R1	−0.168	(0.132)		0.213
	R2	**0.038**	**(0.016)**	*	**0.027**
	PD	0	(0.004)		0.970

*p<0.05.

**Table 7:** Association between qMRI measurements and MS Severity Score (MSSS).

		β	SE	p
NAWM	R1	−2.608	(10.596)	0.807
	R2	−0.848	(0.834)	0.317
	PD	−0.049	(0.375)	0.898
DAWM	R1	2.894	(7.932)	0.718
	R2	0.068	(0.971)	0.945
	PD	−0.042	(0.232)	0.857

### Normal Appearing White Matter

NAWM ROIs could be placed parietally in the centrum semiovale, similarly as placements of NWM ROIs in the healthy subjects.

Statistical analysis revealed that NAWM in the MRI_neg_ group had significantly lower R1 and higher PD than NWM in the healthy subjects. Moreover, NAWM in the MRI_neg_ group had significantly higher PD than in the MRI_pos_ group. NAWM in the MRI_pos_ group, on the other hand, was not significantly different from NWM (for details, see Table 2).

No correlations between qMRI properties of NAWM and clinical parameters were observed in the MRI_neg_ group. In contrast, R1 and R2 in the MRI_pos_ group were negatively correlated to EDSS and MSSS, and these correlations were confirmed when the Spearman correlations were calculated (for details, see Table 3). Furthermore, a significant positive correlation between R1 of NAWM in MRI_pos_ and tCho-Aq concentration was also observed (for details, see Table 4). Correlation analysis of qMRI measurements and qMRS measurements inside the MRS VOIs revealed statistically significant negative correlations between R1 and R2 of NAWM in the MRI_neg_ group, and tNA-Aq concentration. In contrast, such correlations were not observed in the MRI_pos_ group. Positive correlations were also obtained between R1 of NAWM in MRI_pos_ and tCho-Aq concentration. Finally, a positive correlation was detected between R2 of NWM and mIns-Aq, and a negative correlation was detected between PD of NWM and mIns-Aq.

Regression analyses revealed no associations between any qMRI properties of NAWM and BPF or MSSS, when both MS groups were pooled (for details, see Table 6 and Table 7).

### Diffusely Abnormal White Matter

DAWM was mostly detected in periventricular WM, and only rarely in the centrum semiovale. DAWM was observed in proximity to focal WM lesions, but also found in isolation in areas with no visible WM lesions. It was particularly notable that DAWM was detected in the majority of the MRI_neg_ patients with very few or no focal WM lesions. Such DAWM usually extended over large areas, and the border with NAWM was not clearly distinguishable. DAWM ROIs could be placed in the bulk DAWM areas avoiding the borders with NAWM, as has been suggested by Seewann *et al*. [17].

Statistical analysis revealed that DAWM was significantly different from NWM in both MS groups for all qMRI measurements. No statistically significant difference in DAWM was detected between the two MS groups (for details, see Table 2).

When correlating qMRI properties of DAWM with clinical parameters a significant negative correlation was found between R2 of DAWM in the MRI_neg_ group and age. No other significant correlations were detected (for details, see Table 3).

Regression analyses revealed an association between R2 of DAWM and BPF. No similar association was observed between qMRI properties of DAWM and MSSS (for details, see Table 6 and Table 7).

### Re-evaluation of MRIneg

Seven patients in the MRI_neg_ group had developed more than two WM lesions on their present MR examination; therefore all analyses were subsequently re-evaluated, excluding these patients. The time from disease onset to the last MRI-negative scan of these patients was 10±11 years (range 1–36), indicating an atypical period of lesion formation for these subjects.

When the MRI_neg_ group was re-evaluated no differences between the two MS groups reached statistical significance, and the correlation between R2 of DAWM in the MRI_neg_ group with age diminished. This was probably caused by the low number of remaning subjects. However, since the original MRI_neg_ group showed statistically significant differences in qMRI properties, this indicates that the MRI_neg_ group was nevertheless a subgroup suitable to examine MS with atypically low number of focal WM lesions.

## Discussion

We used quantitative magnetic resonance imaging (qMRI) to gain insights on diffuse white matter (WM) changes in two groups of clinically definite (CDMS) patients. One of the MS groups (‘MRI_neg_’) showed very few focal WM lesions in a previous clinical examination, and this group was useful for investigating WM in the absence of focal lesions. The pathological hypotheses presented by Lassman et.al. [39], that include the classification into several subgroups with differing pathogenesis on the cellular level, may have some bearing on such an atypical MS presentation. In this group of patients the clinical investigation must be extraordinarily thorough before a diagnosis of clinically definite (CDMS) can be made, also concerning the radiological investigation [40].

We included normal appearing white matter (NAWM), diffusely abnormal white matter (DAWM), and focal WM lesions in the investigations. Comparisons were performed both between MRI_neg_ and typical MS patients with focal lesions, (‘MRI_pos_’), as well as with healthy controls. Furthermore, correlation analyses were performed between qMRI measurements and a range of clinical parameters, as well as metabolite concentrations in NAWM, measured using quantitative magnetic resonance spectroscopy (qMRS), reported previously in [23]. Finally, regression analyses were performed to determine the associations between qMRI measurements and BPF as well as MSSS.

Several of the MRI_neg_ subjects had developed WM lesions between the clinical examination and the present examination. Even so, these patients were different from the typical MS patients, who exhibited focal lesions early on in the disease. This is supported by the differences found between the MRI_neg_ patients and the typical MS patients. The late presentation of focal lesions rather supported the initial diagnosis of atypical MS.

The qMRI sequence used in this study was optimised to accommodate clinical requirements, in particular regarding the examination time, as well as to simultaneously quantify the three quantitative properties R1, R2 and PD. In order to achieve the short examination time, the speed-up techniques echo planar imaging (EPI) and gradient spin echo (GraSE) were used. This may have lead to some blurring in the R2 maps. Furthermore, off-resonance effects of the saturation pulse may have affected the signal attenuation due to magnetisation transfer. This effect, however, would be systematic and affect all subject groups in similar ways. The slice profiles were accounted for in the parameter estimation by inclusion of a spin model, this was described in detail in [22].

### Normal Appearing White Matter

Changes found in NAWM have previously been attributed to axonal disruption through microglia activation [41], but also to decreased axonal density [19]. In the present study we did not observe any significant difference between NAWM in MRI_pos_ and NWM in healthy controls, but NAWM in the low-lesion MRI_neg_ group was characterized both by significantly higher PD and lower R1 than in normal subjects.

In the MRI_pos_ group it was found that R1 and R2 of NAWM were correlated with EDSS and MSSS. This correlation was not observed in the MRI_neg_ group. Moreover, no association was found between qMRI measurements of NAWM and BPF or MSSS in the regression analysis, when both MS groups were pooled. Conversely, in the previous qMRS study of these subjects, reported in [23], negative correlations were observed between tCr-Aq concentration as well as mIns-Aq concentration and BPF. Also, a positive correlation was then observed between tGlx-Aq concentration and MSSS.

One explanation may be that changes in R1 and R2 as well as changes in BPF indicate different aspects of brain atrophy. However, the relation between tCr-Aq and mIns-Aq with BPF in all subjects may reflect a general atrophy process that is caused by both ageing and MS pathology.

QMRI measurements of NAWM did not show any association with MSSS, whereas our previous qMRS measurements of tGlx-Aq concentration in NAWM did. This suggests that there are pathological processes in NAWM, which could not be detected with qMRI.

A positive correlation was found between the tCho-Aq concentration of NAWM and R1 in MRI_pos_ patients. Since tCho has previously been shown to be a marker for membrane turnover [42] this suggests that R1 may be related to this process. Furthermore, the mIns-Aq concentration of NWM correlated negatively with R2 and PD in the healthy control group. This could be due to an effect of age [24].

The MRS VOIs were large compared to the qMRI ROIs, and mean qMRI values were also measured inside these volumes, and subsequently correlated with qMRS measurements in the same volumes. An interesting observation was that there was a negative correlation between R1 and R2 in the MRS VOIs with tNA-Aq concentration in the MRI_neg_ patients. This indicates that the tNA-Aq concentration in these VOIs actually increased as R1 and R2 decreased. Since tNA is known to be a marker of neuronal density [43] this result suggests that there was a process of demyelination, indicated by decreased R1 and R2, without neuronal loss in these patients. The neuronal density of the tissue (per volume) may in fact increase as myelin diminishes, caused by a net shrinkage of the tissue, leading to more axons inside the VOI. The correlation was not observed in the MRI_pos_ patients, indicating that these patients suffered from axonal loss as well in the NAWM. This could be attributed to Wallerian degeneration, which is considered to be secondary to focal WM lesions [16], [19], [20].

### Diffusely Abnormal White Matter

Previously, changes in DAWM have been attributed mainly to Wallerian degeneration. Seewann *et al.* showed in a comprehensive histopathological study that decreased R1 and R2 in DAWM were associated with axonal loss and decreased myelin [17]. In our present study, DAWM was observed in both the MS group with typical MR presentation, and in the MS group with a low number of focal lesions. Moreover, qMRI properties of DAWM were similar in both groups. Since DAWM was found to a large extent also in the group with a low number of lesions this might suggest that DAWM is not exclusively due to Wallerian degeneration, but that it is also a process in the WM which to some extent is independent of the precense of focal WM lesions.

One major result from this study was that DAWM appears to constitute an intermediary between focal WM lesions and NAWM for all qMRI measurements, in both groups of MS patients. This is consistent with previous findings where Vrenken *et al.* showed that DAWM was an intermediary in T1 [18]. In our view this may reflect the relative increase of the extracellular water of the tissues, where focal lesions have the highest increase, DAWM has a less pronounced increase, and NAWM only has a slight increase. This is supported by previous studies which have shown that decreased R1 and R2 are linked to increased water contents due to increased size of extracellular spaces, caused by a combination of axonal loss and demyelination [16], [17], [25], [26]. This is also consistent with the observed increases of PD. Conversely, the WM lesions detected in the MRI_neg_ group was significantly different from the lesions in the MRI_pos_ group, the lesions in the MRI_neg_ group seems to constitute an intermediate between the MRI_pos_ lesions and DAWM.

Another important finding was that DAWM was common in both groups of MS patients. The MRI_neg_ group had similar disability and clinical presentations as the MRI_pos_ group, even though the number of WM lesions was low and the BPF was unaffected. Furthermore, focal WM abnormalities detected in the MRI_neg_ group were significantly different from the WM lesions detected in the MRI_pos_ group. These abnormalities were more like those in DAWM, and some of them may in fact have been small regions of DAWM misinterpreted as focal lesions.

A correlation was observed between R2 of DAWM in the MRI_neg_ group and subject age, but since this finding was not reproduced in the MRI_pos_ group this might be a coincidental finding. More interestingly an association was found between R2 of DAWM and BPF in the regression analysis. This may be related to increased extracellular water caused by a global inflammation. This should be further investigated in a longitudinal study.

### Limitations of this Study

This study was cross-sectional and as such it was not possible to investigate changes of qMRI properties over time, nor the relation to disease progression. Nevertheless, associations between some of the qMRI measurements and clinical parameters as well as metabolite concentrations were observed, and these findings should be verified in a longitudinal study.

In total, 168 correlation coefficients were calculated; by chance 5% of these would fall out as significant (p<0.05). Therefore, caution is warranted when interpreting the results and some of the findings may have been caused by the effect of multiple comparisons. The findings in this study should be investigated and confirmed in larger population based studies.

Furthermore, other studies have highlighted the multi-exponential behaviour of T2 in WM [44], [45], and recently, the multi-exponential behaviour of T1 has been reported [46]. MacKay *et al.* carried out work with a 32 echo CPMG sequence in order to directly measure the sizes of the different water compartments, and in this way reach a surrogate measurement of myelin solids in the tissue [45]. In the present study a fast qMRI sequence capable of measuring a single component of R1, R2 and PD in the same scan was applied. Although this sequence measured all three properties in the same scan, only one component for R1 and R2 was measured. In this way the different compartments were averaged together and it was not possible to establish whether decreased R1 and R2 rates were due to loss of myelin, increased intra/extra-cellular water, or inflammation.

## Conclusions

A major finding of this study was that DAWM appears to constitute an intermediate between focal WM lesions and NAWM in terms of qMRI properties. Remarkably, the MRI_neg_ group exhibited similar amounts of DAWM as the MRI_pos_ group, with similar qMRI characteristics. This was the case even though WM lesions were not common and BPF was unaffected in the MRI_neg_ group. Furthermore, qMRI measurements on NAWM in the MRI_neg_ group were correlated to qMRS measurements of tNA-Aq concentration, suggesting that these patients suffered demyelination without axonal loss. The results suggest that changes in NAWM and DAWM can be caused by pathological processes, and are not only due to Wallerian degeneration and focal WM lesions. QMRI measurements of NAWM and DAWM may provide important markers for the disease status. In particular, the correlation between qMRI properties and EDSS as well as MSSS may shed some new light on the clinicoradiological paradox, although these results should be interpreted with caution due to the limited number of patients.
